# Development of Ordered, Porous (Sub-25 nm Dimensions) Surface Membrane Structures Using a Block Copolymer Approach

**DOI:** 10.1038/s41598-018-25446-0

**Published:** 2018-05-08

**Authors:** Tandra Ghoshal, Justin D. Holmes, Michael A. Morris

**Affiliations:** 1 School of Chemistry and Tyndall National Institute, University College Cork, Cork, Ireland; 2 AMBER and Department of Chemistry, Trinity College Dublin, Dublin, Ireland

## Abstract

In an effort to develop block copolymer lithography to create high aspect vertical pore arrangements in a substrate surface we have used a microphase separated poly(ethylene oxide) -b- polystyrene (PEO-b-PS) block copolymer (BCP) thin film where (and most unusually) PS not PEO is the cylinder forming phase and PEO is the majority block. Compared to previous work, we can amplify etch contrast by inclusion of hard mask material into the matrix block allowing the cylinder polymer to be removed and the exposed substrate subject to deep etching thereby generating uniform, arranged, sub-25 nm cylindrical nanopore arrays. Briefly, selective metal ion inclusion into the PEO matrix and subsequent processing (etch/modification) was applied for creating iron oxide nanohole arrays. The oxide nanoholes (22 nm diameter) were cylindrical, uniform diameter and mimics the original BCP nanopatterns. The oxide nanohole network is demonstrated as a resistant mask to fabricate ultra dense, well ordered, good sidewall profile silicon nanopore arrays on substrate surface through the pattern transfer approach. The Si nanopores have uniform diameter and smooth sidewalls throughout their depth. The depth of the porous structure can be controlled via the etch process.

## Introduction

There is great interest in the fabrication of nanoporous arrays in silicon and other similar material substrates arrays. Here, we define these at substrate surfaces containing nanopores (<100 nm diameter) that are vertical to the surface plane. The interest is justified by the wide range of potential applications in advanced optoelectronics, nanophotonics, microelectromechanical systems and biomedical application^1–6^. However, the fabrication is compatible with existing Si-based semiconductor processing technologies, this becomes progressively more expensive and technically challenging as feature size decreases and costs are incompatible with the application area. Thus, fabrication of nanoporous substrates (particularly of controlled arrangement, orientation and pore dimension) that are cost-effective and suitable for large volume manufacturing production is a significant challenge. Many nanopatterning techniques were utilized to produce highly ordered nanopore structures on Si substrate but have important disadvantages. Methods including nanosphere lithography (defect density, lower feature size limitations, surface density of pores), electron beam lithography (cost and large volume production), metal assisted chemical etching (pore size, controllability), focussed ion beam (size, cost, large volume) etc^7–11^. In general, these processes are costly because sophisticated instruments and complicated procedures are needed and limited to the feature size and time.

In comparison, films of self assembled diblock copolymers (BCPs) have attracted significant attention for the generation of nanopore arrays of sizes below 100 nm in a relatively simple and efficient manner with low cost through the etching process. Spin coating and solvent annealing is a proficient method where BCP ordering can be accomplished within small time periods under solvent vapour atmosphere because of occurrence of polymer swelling by the solvent to create free volume and provide sufficient chain mobility and rearrangements to the polymer blocks to assist self-assembly^12,13^. The methods by which BCP patterns can be created and used for pattern transfer are described elsewhere^14^. A challenging abstract is often control over microdomain orientation and/or lateral ordering which is dictated by a complex interaction of surface energies, entropic factors, polymer-solvent interactions, and the commensurability between the film thickness and domain spacing^15,16^. In context, polystyrene-*b*-polymethylmethacrylate (PS-*b*-PMMA) has been the greatest system of interest extensively studied because of its accessibility, ease of production, and well-established processing technologies^17–19^. Despite significant advancement, there are limitations such as long processing times^20^, controlling domain orientation^21^ and minimum feature size achieved^22^. A further challenge is the pattern transfer of the cylindrical phase system to the substrate because of limitation of the etch contrast between the blocks and combined with the small diameter of cylinders, poor shape control can result^23–25^. Various methods have been used to enhance etch contrast including organic and inorganic features into one of the microdomain^14,26,27^. We have developed a simple solution mediated metal ion (Fe, Ni, Zn etc.) insertion by using PS-PEO BCP where chemical co-ordination between metal ion and PEO microdomains produces inorganic patterns that can act as ‘hard’ masks to allow facile and efficient, high aspect ratio pattern transfer into Si substrate^27–34^. In comparison, iron oxide is acting as an excellent hard mask over silicon^27,29,31,32,34^. However, in common with much of the other BCP work (such as PS-P4VP and PS-P2VP etc.), the cylinders were reconstructed (not etched) for the metal ion insertion and nanowires or nanorods are produced rather than well-defined pores^35,36^.

Herein, we extend our previous work towards developing an innovative, simple methodology for creating a nanoporous etch mask to fabricate high aspect ratio nanoporous Si patterns using PEO-b-PS BCP (PEO the majority phase) through a reactive ion etch process. PEO-b-PS microphase separation is demonstrated by tuning solvent annealing parameters, namely, solvents, temperature, time etc. to create vertically (to surface plane) orientated PS cylinders in PEO matrix. This is then used to create an inorganic etch mask to form good sidewall profile, uniform well ordered Si nanopore network through a plasma based pattern transfer process.

## Results

### Microphase separation of PEO-b-PS thin films

**Figure 1:**
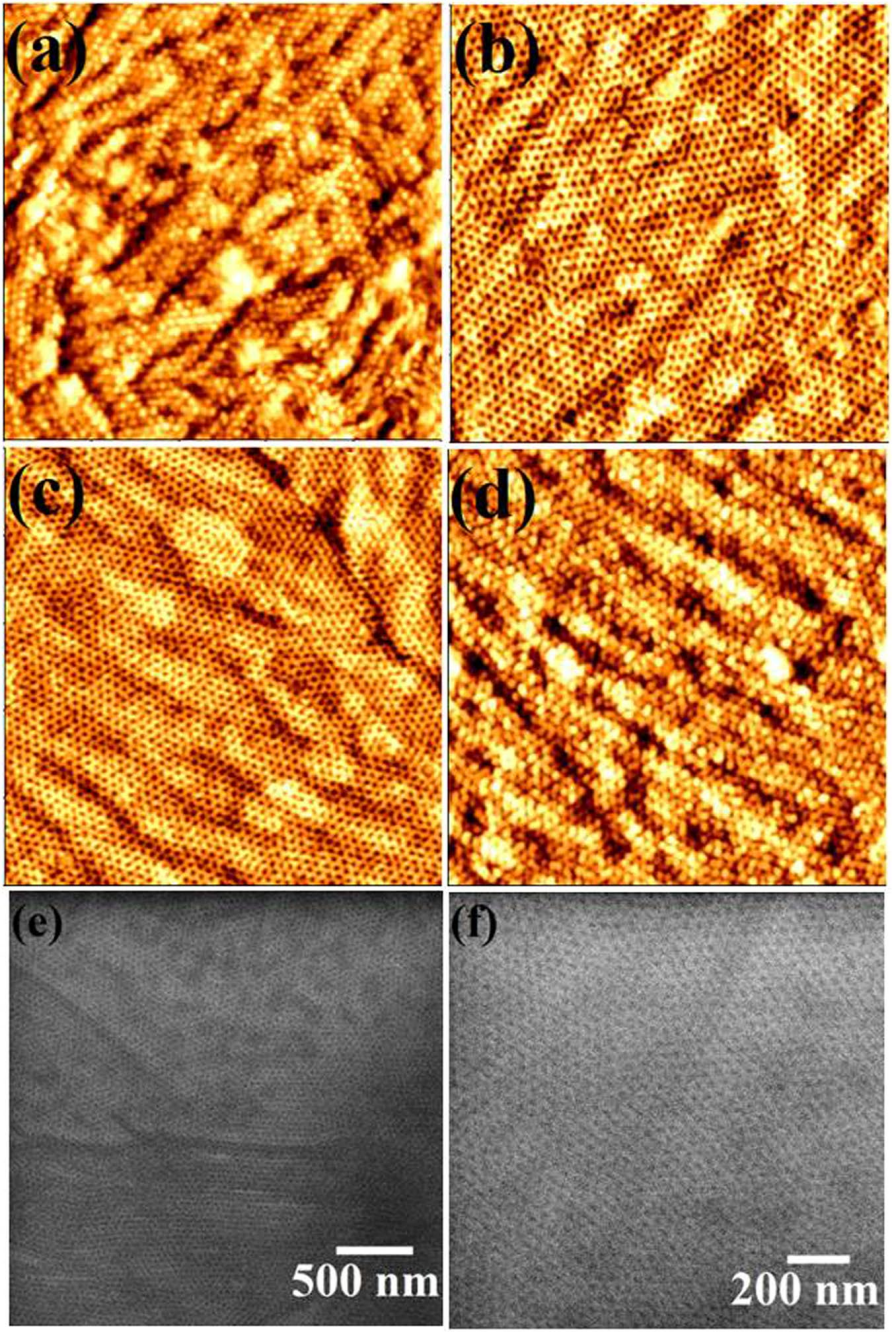
AFM images (2 × 2 μm) of hexagonal ordered PEO-b-PS thin film following solvent annealing in Chloroform at different temperature of (**a**) 40 °C, (**b**) 50 °C, (**c**) 60 °C and (**d**) 70 °C for 30 min. (**e**,**f**) SEM images of the film at 60 °C.

The BCP studied here is Poly(ethylene oxide)-b-Polystyrene (PEO-b-PS) (39.5k-16.4k) with PS as the cylinder forming minority composition microdomain. To control and achieve the perpendicular orientated cylindrical structures, annealing solvent, temperature and time are carefully calibrated. Similarly prepared BCP thin films have been placed in the annealing chamber with different annealing solvents, time and temperature in order to achieve long range ordered nanopatterns. The optimum ordering of the BCP showing the perpendicular orientation of PS cylinders inside PEO matrix was achieved by annealing the BCP films under Chloroform (CHCl_3_) vapour for 30 min. Increasing the solvent annealing time resulted in poorer long range ordering, disordered regions and thickness variation are observed (not shown). The annealing temperature was varied and the surface morphologies of the PEO-b-PS thin film after solvent annealing in Chloroform at different temperature for 30 min are shown in (Fig. 1). As-cast films possess a disordered morphology without any indication of feature periodicity. Solvent annealing results in the formation of well ordered arrangement of perpendicularly oriented PS cylinders in PEO matrix (PS cylinders are darker in the AFM image). Note that thermal annealing at the same temperature in the absence of the solvent has no effect on the as-cast disordered structure demonstrating that Chloroform is a necessary component to induce phase separation. At lower temperature at 40 °C (Fig. 1a), the surface film displays a quasi-hexagonal micellar structure but the film is noticeably rough obvious regions of multiple micelle layers. This morphology may represent a disordered, intermediate stage driven by the interplay between microphase separation and dewetting. At this temperature, we suggest the film does not have enough mobility to form the cylindrical microphase separated arrangement. The size and density of the micellar structure decreases with increasing the annealing temperature and microphase separation becomes obvious as micelles are converted towards the extended well-ordered structure. This is obvious from data at annealing temperatures of 50 °C (Fig. 1b) and 60 °C (Fig. 1c) as the surface is smooth and the domain structure extends across the film. The hexagonal ordering enhanced at higher temperature of 60 °C. The SEM images shown in (Fig. 1e,f) also depicts the hexagonal well ordered nanopatterns. The centre to centre cylinder distance measured by AFM and SEM is 52 nm whereas the cylinder diameter is 22 nm. The film thickness measured by ellipsometry is 35 nm. Further increase in the annealing temperature to 70 °C and above results in loss of hexagonal ordering (one can clearly see regions of phase separated structure and regions of micelles) and increase the surface roughness (Fig. 1d). 70 °C is above the CHCl_3_. boiling point and we would suggest that swelling is so extensive that during solvent exposure micelles can form in a pseudo solution.

Note that the PEO-b-PS systems dictate asymmetric affinities for both the substrate-polymer and the polymer-air interfaces. Previous studies advocate preferential wetting of the substrate surface for the hydrophilic PEO whilst PS segregates to the air interface (lower surface energy of PS than PEO, γ_PS_ = 33 mNm^−1^; γ_PEO_ = 43 mNm^−1^)^33^. The volume fraction of PS used in this work is 0.297 suggest the formation of PS cylinders. We have also studied the effects of microphase separation process and the surface morphologies by varying the PS/PEO ratio; achieved by blending the BCP with either PS homopolymer (mol. Wt. 16000) or PEO homopolymer (mol. Wt. 40000) in 9:1 ratio (see Supporting Information). For PS-b-PEO (i.e. the reverse composition), toluene can be used as a suitable solvent since it is a good solvent for the glassy PS matrix. Toluene is highly selective for PS compared to PEO because of smaller solubility parameter difference with PS (δ_Tol_ − δ_PS_ = 18.3-18 = 0.3 MPa^1/2^) than with PEO (δ_Tol_ − δ_PEO_ = 18.3–20.2 = 1.9 MPa^1/2^). For the inverse system studied here, toluene based solvent annealing is not possible because of the low affinity for the EO type matrix. The solvent anneal temperature was set at 60° C (boiling point of Chloroform is 61° C) in order to endorse diffusion of the annealing solvent chloroform through the entire film thickness which also leading to a higher evaporation rate of chloroform. It is clear that at 60 °C, the CHCl_3_ is a good solvent and swelling and mobility is induced so as to form phase separated films in 30 min. Upon exposure to Chloroform vapour, both PS and PEO chains moves and extend because of non selective nature of the vapour (δ_chlo_ − δ_PS_ = 18.8-18 = 0.8 MPa^1/2^, δ_chlo_ − δ_PEO_ = 18.8–20.2 = 1.4 MPa^1/2^). This depicts the simplicity and ease of the system re-orientation to achieve microphase separated nanopatterns for short annealing time.

### Schematic description of fabrication of Si nanopore arrays

**Figure 2:**
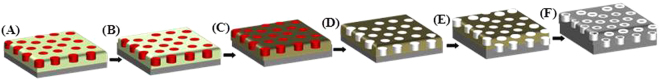
(**A**) Solvent annealing of the spin coated film in Chloroform produces hexagonally arranged PS cylinders perpendicular to the substrate in PEO matrix, (**B**) Modification of PEO matrix creates nanoporous template where PS cylinders exposed to the film-air interface, (**C**) Iron oxide precursor solution spin coated onto the template, (**D**) Iron oxide nanohole arrays were prepared by UV/ozone treatment, (**E**) Si nanopores with iron oxide at top were fabricated by consecutive silica and silicon ICP etch processes, (**F**) Ordered Si nanopore arrays were formed after removal of oxide mask.

The BCP nanopatterned templates can be used to generate good sidewall profile Si nanopore arrays through the hard mask pattern transfer process. Our previous works suggests that^29,31,32,34^ iron oxide nanodots and nanowires can be used as a resist mask over silicon to create high aspect ratio well ordered substrate features. Silicon nanopore arrays generated with a similar strategy as described in (Fig. 2). Spin coated BCP film upon solvent annealing in Chloroform produces hexagonally patterned PS cylinders orientated perpendicular to the substrate inside PEO matrix (Fig. 2A). Chemical etching and/or modification of PEO matrix results nanoporous template where PS cylinders exposed to the film-air interface (Fig. 2B). Iron oxide nanohole arrays were generated by spin coating metal ion precursor-alcohol solution (Fig. 2C) followed by UV/ozone treatment which oxidize the precursor and remove the polymer blocks (Fig. 2D). Si nanopore arrays with iron oxide matrix were fabricated by successive silica and silicon ICP dry etch processes (Fig. 2E). Ordered Si nanopore arrays were formed after removal of oxide mask (Fig. 2F).

### Fabrication of BCP nanopatterned template

**Figure 3:**
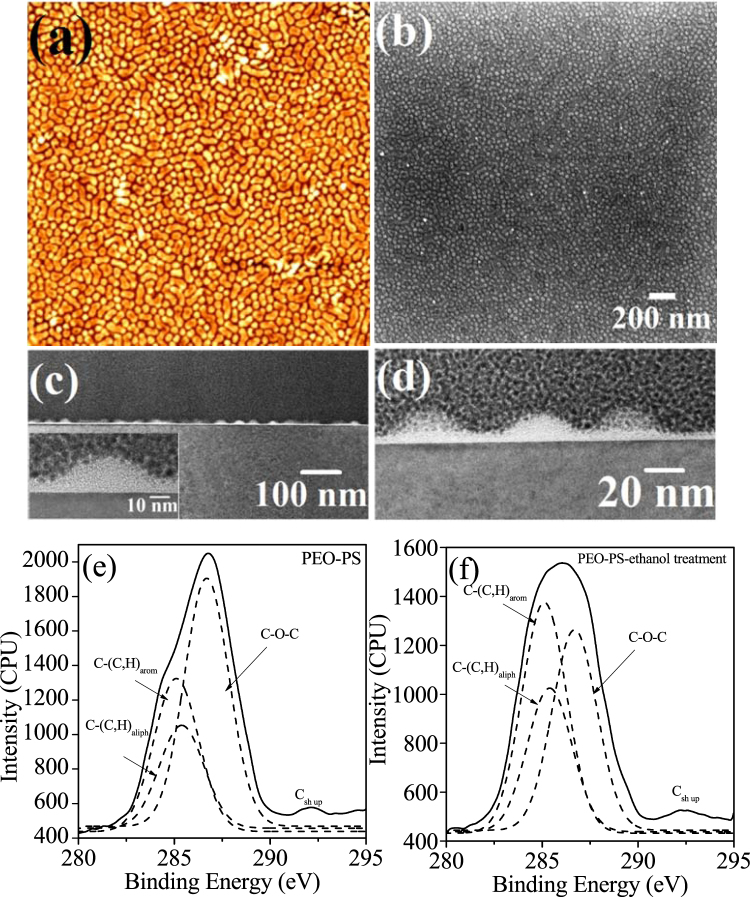
(**a**) AFM (2 × 2 μm) (**b**) SEM and (**c** and **d**) cross-sectional TEM images of hexagonal ordered nanoporous network after chemical etching/modification of PEO matrix by anhydrous ethanol. XPS C1s spectra of the thin films (**e**) before and (**f**) after ethanol treatment.

It is required to ‘activate’ the PEO which removes most of the PEO and densified with a thin layer of PEO as reported earlier for hexagonally arranged PS-b-PEO system, to produce nanopatterned template^28,31–34^. Chemical etching and/or modification of the PEO matrix were achieved by immersing the film at 40 °C in anhydrous ethanol since ethanol is non-interactive solvent to PS. (Fig. 3a,b) shows the AFM and SEM images of the PEO-PS thin film (prepared at 60 °C for 30 min) after ethanol exposure for 16 h. The feature size and periodicity (cylinder spacing = 52 nm and PS cylinder diameter = 22 nm) of the BCP patterned template remained unaltered through this process. The film thickness decreases to 22 nm as measured by ellipsometry which means the ethanol treatment partially densifies or degrade the PEO matrix. The result of the activation is to generate a topographical profile with the PS cylinders proud of the remaining, continuous EO matrix as clearly shown in AFM and SEM images. No dewetting or missing patterns were observed for the nanoporous template.

### Morphology and interface of BCP template by cross-sectional TEM

Cross-sectional FIB thinned TEM helps to verify the structural integrity and ordering of the nanoporus film template (Fig. 3c,d). The comparable densities of the PS (1.05 g cm^−3^) and amorphous PEO (1.12 g cm^−3^) results in undistinguished blocks in TEM micrographs for the PEO-PS micrphase separated films. This suggests that no PEO crystallization occurred during solvent annealing which could provide TEM contrast since the crystalline PEO is denser (1.24 g cm^−3^)^37,38^. TEM image in (Fig. 3c) shows a continuous nanoporous template on Si substrate without any deformation at the interface. Higher magnification images (insets of Fig. 3c,d) describes the average diameter of PS cylinders of about 22 nm consistent with the surface profile and suggesting no significant strain on the cylinders at the interfaces. The thickness of the film i.e. the height of the PS cylinders is approximately 22 nm whereas the thickness of the PEO matrix decreases to 5 nm after ethanol treatment.

### XPS analysis of BCP nanopatterns and template

The films before and after ethanol treatment were analysed by XPS to further study the activation process. The C1s peaks were curve-fitted to reveal four components as shown in (Fig. 3e,f). The peak at 284.9 eV and 285.3 eV can be recognized as carbon aromatic ring of PS (**C**-(C,H)_arom_) and the aliphatic backbone of PS (**C**-(C,H)_aliph_) respectively. A distinct high binding energy C1s shoulder peak at about 286.6 eV is acknowledged particularly for the microphase separated film and assigned to carbon atoms in the ether link (**C**-O-**C**) of PEO. A shake-up satellite at about 291.8 eV assigned to the aromatic ring of PS (**C**_sh up_) exists for both films. The contribution of PEO component decreases from 70% to 12% after the ethanol treatment (measured from total C1s peak area including adventitious signal), consistent with most of the PEO removal but possibility of some of the PEO remains in its crystalline form.

Here, ethanol was preferred as PEO removal solvent because of similar solubility parameters, vapour pressure and chemistry. Further, similarity in the chemical structures of PEO monomers [(CH_2_CH_2_O)-] and ethanol molecules (H-CH_2_CH_2_O-H) is also equally important. Most of the PEO fragments were dissolved in ethanol. Ethanol also an ideal solvent to allow crystallization of the PEO at a certain temperature^33^. The film should be taken out the film from the solution and dried to avoid film swelling or thickness variation. After the desired time to ethanol exposure, PEO molecules prefers to separate from the solution at room temperature but PEO monomers and ethanol molecules cannot distinguish themselves from each other, thus forming a thin crystalline layer of PEO whereas some of ethanol molecules were trapped^39^.

### Generation of inorganic oxide hard mask nanopatterns

**Figure 4:**
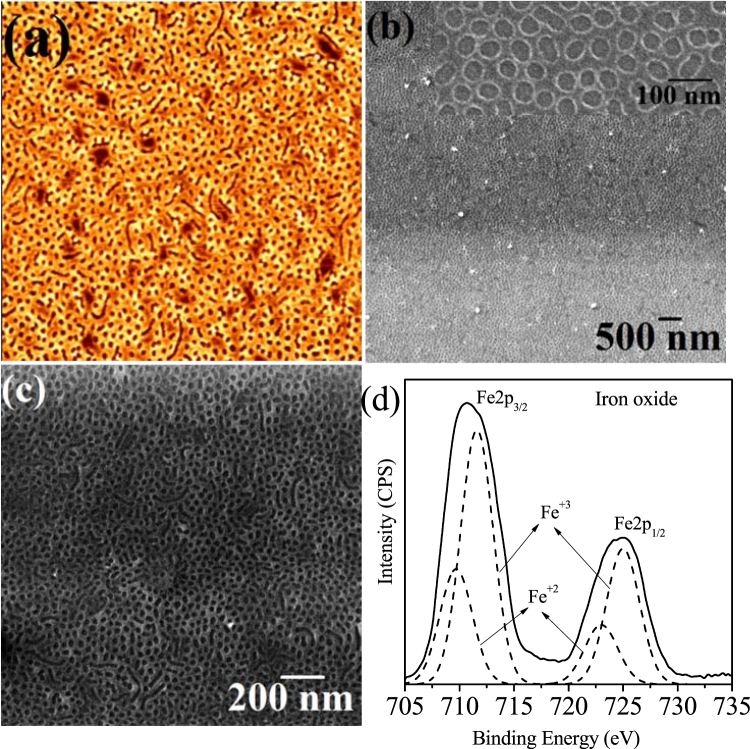
(**a**) AFM (2 × 2 μm) (**b**,**c**) SEM images of hexagonal ordered nanoporous network prepared by spin coating the metal ion precursor solution followed by UV/ozone treatment. (**d**) XPS Fe 2p spectra of the as-prepared iron oxide nanopores.

The polymer nanoporous template can be used to achieve similarly ordered iron oxide nanohole arrays by the metal ion inclusion method described above. (Fig. 4(a–c)) represents the AFM and SEM images of the iron oxide nanoholes prepared by spin coating 0.3 wt% precursor-alcohol solutions onto the nanoporous film. Here, anhydrous 2-propanol is chosen to dissolve the iron precursor because of its selective solubility nature into PEO (similar solubility parameters δ_PEO_ = 20.2 MPa^1/2^, δ_2propanol_ = 23.5 MPa^1/2^). Similar treatment with ethanol causes undulation, pattern degradation and swelling between nanoholes (See Supporting information). Ethanol molecules might also have a deleterious impact because of their reaction with the PEO blocks as described above^32,34^. Metal ion inclusion into the PS cylinders is hindered because of its hydrophobic nature and the affinity of PEO with the ionic solution selectively incorporate the ions through either intra- or intermolecular coordination via electron donation from the PEO block with the oxygen species in the 2-propanol molecule^33^. We cannot rule out the contribution of the inclusion process to phenomena such as wetting and the rate of the inclusion reaction^40^. There is a gain in the free energy of the process derived from the interaction between PEO and metal cations which create an additional driving force for the inclusion. (Fig. 4a) clearly shows iron oxide ordered nanohole film on the substrate. Swelling between holes and pattern degradation is evident in few areas. The SEM images in (Fig. 4b,c) indicates the long range ordering of the nanoholes over the coupons used here. The diameter of the of the iron oxide ‘holes’ in the film varied between 22–25 nm with a spacing of 52 nm in agreement with the original template size and spacing. Note that the concentrations of the iron precursor solutions were adjusted to form uniform, continuous and smooth nanhole patterns. Higher concentrations lead to overfilling of the pores and secondary overlayer formation whereas less concentration forms discrete nanoholes (See Supporting information).

It is also important to filtrate the coating solution. The larger (>200 nm) particles were filtrated while smaller particles remains within the precursor solution. The particles become agglomerated and thus increased in size (van der Wals forces) with stirring time and eventually these become large enough to restrict the ordered arrangements. Thus, filtration is a necessary step for the formation of nearly defect free ordered arrangements.

Defects in the form of thickness undulation or small particles on the film surface can also be seen. This generally occurs due to spin coating involving convection of the coating solution, driven by centrifugal force and evaporation of the solvent simultaneously^41^. Spin coating results some of the residual solvent reside on the film surface in the form of droplets because of surface tension effects and also due to the slower evaporation rate of 2-propanol (some amount of water present). Unreacted metal ions within the droplet were oxidized by the Uv/Ozone treatment. A higher metal precursor concentration might result overloading of material (See Supporting information) consistent with the proposed preferential PEO-solution inclusion model whereas simple template mechanism forms non-specific surface depositions throughout the solution concentration range^42^.

### Chemical composition of inorganic oxide by XPS

The chemical composition of the surface of the iron oxide nanoholes was studied by Fe 2p high resolution XPS spectrum (Fig. 4d). The Fe 2p core level spectrum of the iron oxide nanoholes consists of two broad peaks at 711.1 eV (Fe 2p_3/2_) and 724.5 eV (Fe 2p_1/2_) suggests the existence of both the Fe^+2^ and Fe^+3^ ions. The peak was fitted using Gaussian-Lorentzian line shapes provides individual binding energies at 709.6/722.9 eV assigned to Fe^+2^ and 711.5/724.9 eV to Fe^+3 ^^43^. The calculated total peak area ratio Fe^+3^/Fe^+2^ was estimated to about 2:1 as expected for Fe_3_O_4_.

### Interface and composition by cross-sectional TEM and EDAX mapping

**Figure 5:**
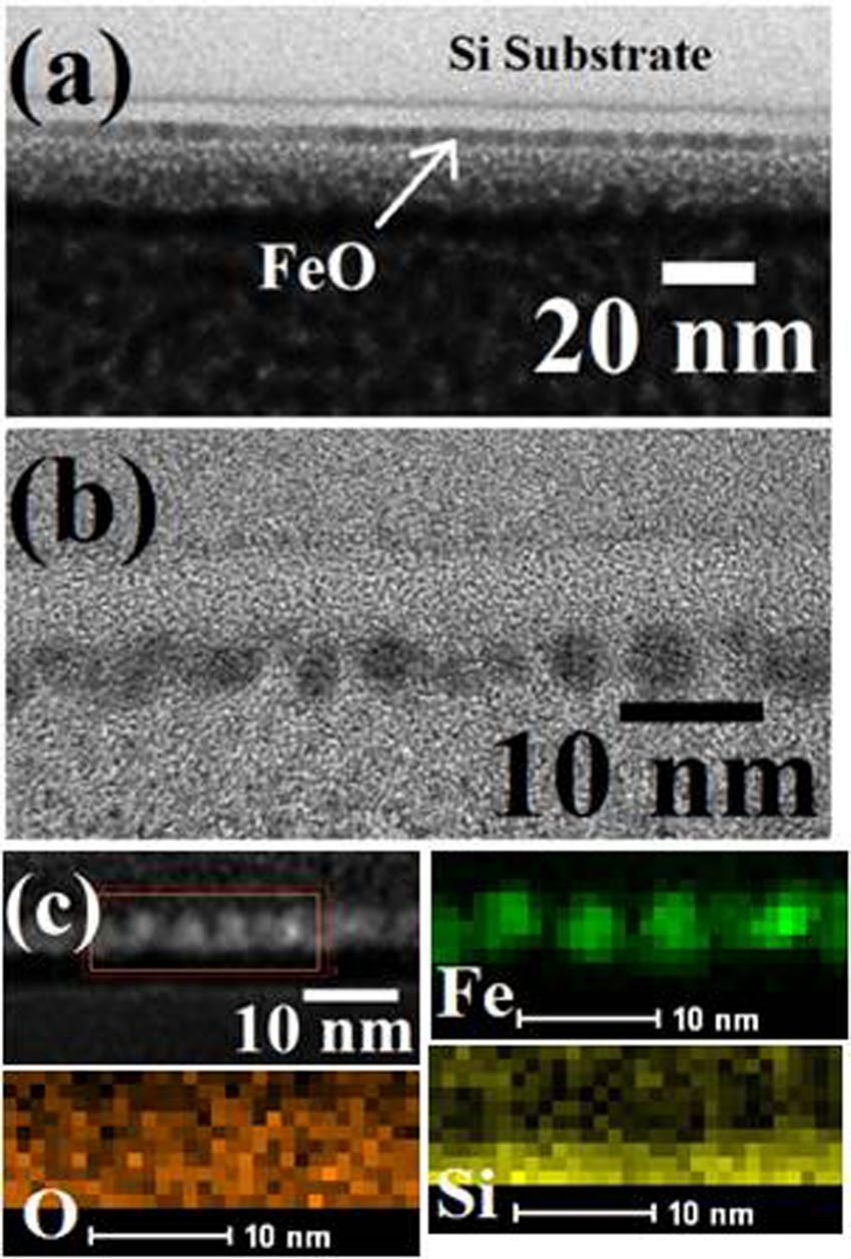
(**a**,**b**) Cross-sectional TEM images hexagonal ordered nanoporous iron oxide network. (**c**) represents corresponding elemental mapping for Fe O, Si.

The structural quality, composition and the substrate-iron oxide film interfaces were analysed using FIB-thinned TEM cross-sections. The interface of the porous film is rigid with the substrate surface without any interfacial cracks occurred during FIB processing reflects the adhesion and structural integrity of the film. This approach is advantageous over lithographic patterning of a oxide film where delamination and mechanical damage under strain results due to interfacial tensions^44^. The cross-sectional TEM image shows the morphology of the film sectioned through the hole structure (Fig. 5a,b). The thickness of the film is ~8 nm. High resolution EDAX mapping confirms the elemental composition and the distribution of Fe, O and Si component shown in (Fig. 5c). The Fe map corresponds to homogeneous distribution of iron for the nanoporous film and a sharp elemental interface with the substrate surface suggesting no iron ion diffusion within the substrate occurs. The O and Si maps confirm the presence of oxides on the film and native layer of the substrate.

### Fabrication of Si nanopore arrays

**Figure 6:**
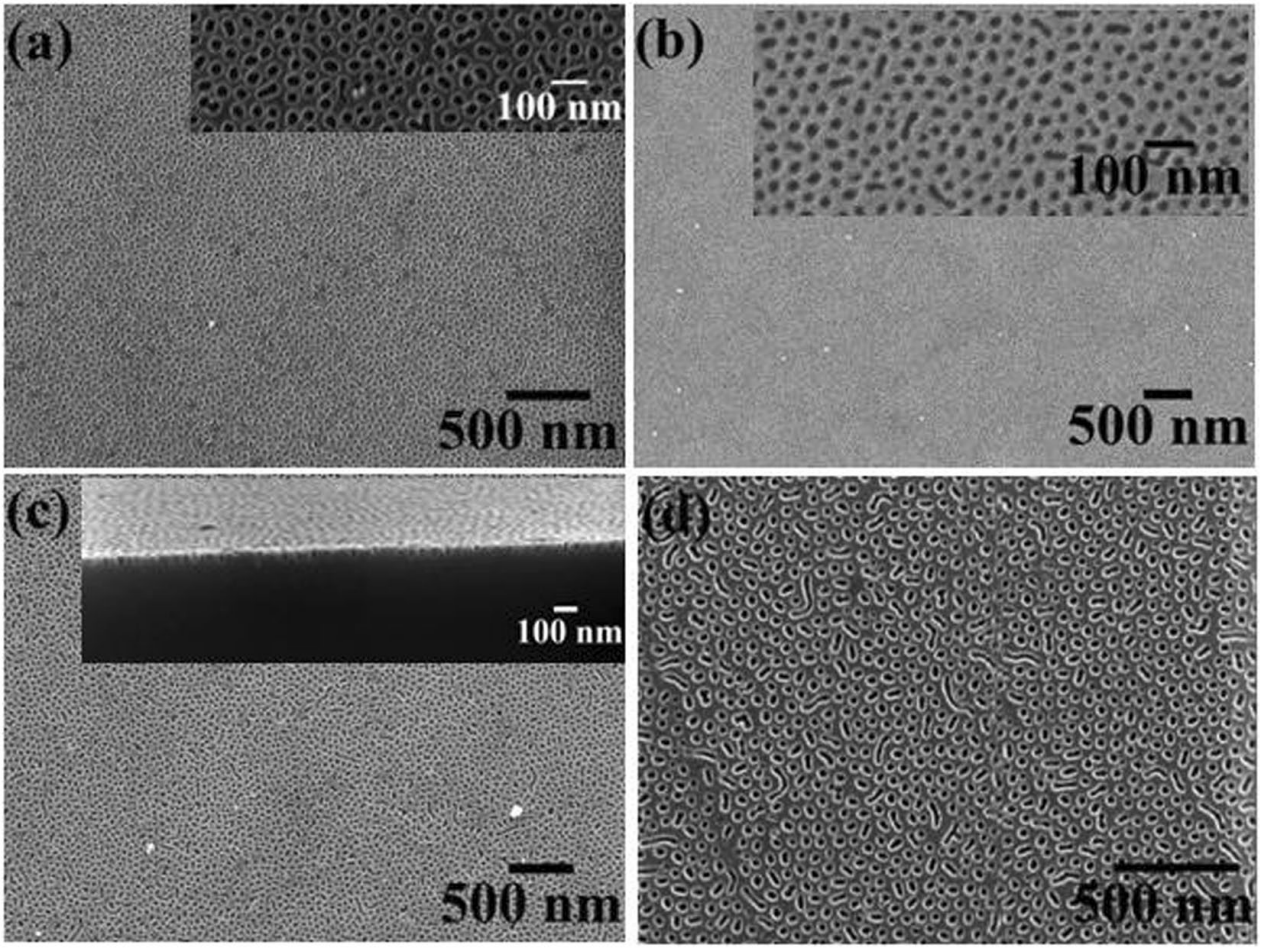
SEM images hexagonal ordered nanoporous Si patterns formed by pattern transfer for different etch time (**a**) 1 min, (**b**) 2 min, (**c**) 3 min, and (**d**) 4 min.

In order to fabricate Si nanopore patterns on the substrate surface, the iron oxide nanohole film were used as a hardmask in the ICP dry etch process. A gentle oxalic acid aqueous solution is efficient to eliminate undesired Fe_3_O_4_ from the Si nanopore arrays. (Fig. 6(a–d)) shows SEM images of Si nanopores over large substrate areas following a 1 min, 2 min, 3 min and 4 min Si etch. The contrast enhancement clearly reflects the pattern transfer into the substrate. Higher magnification images (insets of Fig. 6a,b) demonstrate that the centre to centre spacing remained unaltered whereas the pore diameter varied from 22–25 nm. This suggests no significant damage the original ‘mask’ during pattern transfer. The depth of the Si nanopores increases by increasing the Si etch time confirmed from gradual SEM contrast enhancement. The cross-sectional SEM data give approximate nanopore depth of 60 nm for 3 min etch. All the images reveal large scale fabrication of ordered continuous Si nanoporous film.

### Interface and composition of Si nanopore arrays by TEM and EDAX mapping

**Figure 7:**
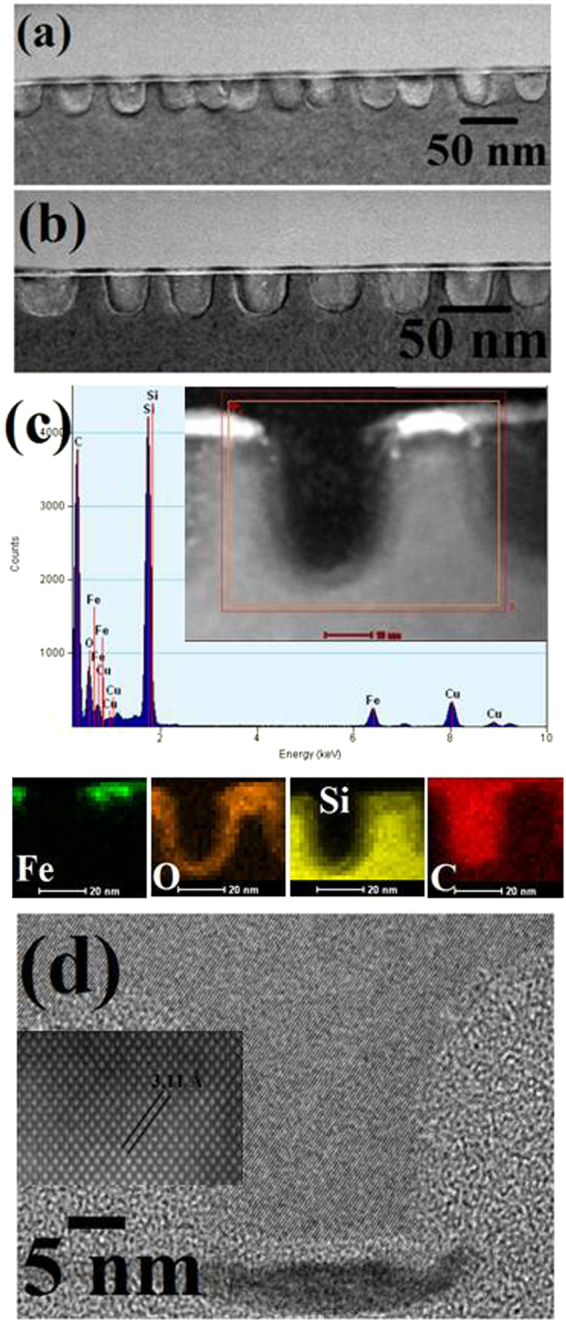
(**a**,**b**) Cross-sectional TEM images hexagonal ordered nanoporous Si pattern for the 2 min etch time. (**c**) EDAX and corresponding elemental mapping for Fe O, Si (**d**) HRTEM image of Si nanopore.

A detail assessment of the pattern transfer process is examined using TEM cross-sections. The morphology, interface, crystallinity of the Si nanoporous patterns as well as an estimation of the mask erosion rate is studied. (Fig. 7a,b) show micrographs of the hexagonal ordered nanopore array of 2 min etched nanopores on Si substrates. The higher magnification image (Fig. 7b) reveal equidistant (~52 nm) Si nanopores of about 40 nm depth and 22 nm diameter with a 8 nm mask layer on top of the film. These data reflects the rigidity of the mask in the etch conditions applied. The quality of the sidewalls such as roughness, amorphortization etc. was also revealed by the higher magnification TEM image. As can be seen, the diameter of the nanopores (~22 nm) remained unaltered along their length. The mask and its’ structural integrity is also reflected through the FIB processing. Elemental composition by high resolution EDAX mapping shows the compact allocation of Fe, O and Si shown in (Fig. 7c). The Si and Fe map shows a homogeneous distribution of silicon corresponding to the nanopore sidewall with a distinct elemental interface with iron oxide suggesting no iron ion diffusion within the substrate occurs after pattern transfer. The O map confirms the presence of oxides in iron oxide at top and native silica layer of the substrate. Importantly, the HRTEM image (Fig. 7d) of the substrate reveals a highly crystalline structure reveals no amorphortization occurred during pattern transfer. The lattice fringes are continuous between bulk and nanopore silicon sidewall indicating absence of any stacking or other defects and hence no re-crystallization during etching. Inset of (Fig. 7d) shows the lattice fringe spacing of 3.11 Ǻ across the nanopore wall agrees well with Si fcc (111) interplanar distances^45^. The (111) fringes makes 54° angle with the substrate surface plane consistent with the (100) orientation of the Si wafer. Thus, the fabricated Si nanoporous patterns on Si substrate were exclusively following the original mask patterns.

In summary, we present large scale fabrication of high density, uniform, hexagonal Si nanopore arrays with precise placement on substrate based on insitu hard mask microphase separated BCP inclusion approach in a simple and cost effective way. Hexagonally arranged self-assembled PEO-b-PS nanopatterns with perpendicularly oriented PS cylinders inside PEO matrix was realized by a simple solvent annealing process as a function of annealing temperature. An effective ethanol treatment was followed for etch and/or modification of the PEO matrix to create nanoporous templates for the generation of inorganic oxides without pattern damage. A selective metal ion inclusion approach followed by UV/Ozone treatment was applied for generating oxide nanohole arrays without any pattern degradation/damage. The nanoholes are structurally arranged in a mimic of the original self-assembled BCP pattern and show strong adherence to the substrate. Large area, identical ordered, Si nanopore (diameter ~ 22 nm) arrays is fabricated with a smooth sidewall profile by using these iron oxide nanoholes as a hard mask over silicon. The nanopores observed were crystalline with desirable uni-axial crystallographic orientation. Further the depth of the nanopore can be varied by varying the etching time. No additional defects, inter-diffusion or amorphization of the materials was observed during the pattern transfer process. This self-assembled hardmask nanolithography can also be an important component in the nanoscale devices manufacturing industry with high throughput and low cost. We suggest this may be a novel method to create porous substrate surfaces and membranes and is applicable in numerous applications.

## Experimental

### Generation of iron oxide nanohole arrays by block copolymers

Poly(ethylene oxide)-b-Polystyrene (PEO-b-PS) diblock copolymer was purchased from Polymer Source Inc. and used without further purification (number-average molecular weight, *M*_n_, PEO = 39.5 kg mol^−1^, *M*_n_, PS = 16.4 kg mol^−1^, *M*_w_/*M*_n_ = 1.06, *M*_w_: weight-average molecular weight). Single crystal, B doped P type silicon (100) wafers with a native oxide layer were used as a substrate. Substrates were cleaned by acetone and toluene for 30 minutes each through ultrasonication and dried under nitrogen stream. PEO-b-PS was dissolved in toluene to obtain 1 wt% polymer solution which was aged for at least 12 h at room temperature. The PEO-b-PS thin film was formed by spin coating the polymer solution (3000 rpm for 30 s). The films were placed at the bottom of a closed vessel kept at different temperature for different time period under Chloroform vapour to induce necessary mobility within copolymer blocks and allow microphase separation to occur. Partial chemical etching and/or modification of PEO matrix was carried out by immersing the film at 40 °C for 16 h in anhydrous alcohol^28,32,33^. The films were taken out from alcohol after the desired time and dried immediately. For the fabrication of iron oxide nanopores, Iron (III) nitrate nonahydrate (Fe(NO_3_)_3_,9H_2_O) was dissolved in anhydrous 2-propanol and spin-coated onto the activated nanoporous template^29,30^. UV/Ozone treatment was used to oxidize the precursor and remove polymer.

### Pattern transfer using Inductively Coupled Plasma (ICP) etch

These iron oxide nanohole arrays were used as a hard mask for pattern transfer to the silicon substrate using an STS, Advanced Oxide Etch (AOE) ICP etcher as previously reported with the nanodot arrays^31^. The system has two different RF generators, one, to generate and control the plasma density by direct connection to the antenna coil, while the other one was used to adjust and control the energy of the ions by connecting it to the substrate holder. A double etching process was used to, firstly, etch the thin native silica layer and, secondly, the silicon substrate. During etching, the sample is thermally bonded to a cooled chuck (10 °C) with a pressure 9.5 Torr. The process parameters were optimised to a C_4_F_8_/H_2_ gas mixture (21 sccm/30 sccm) using an ICP coil power of 800 W and a Reactive Ion Etching (RIE) power of 80 W for the oxide layer etch. The silica etch time was kept constant (5 sec) for all the samples. For Si pore fabrication, the process used a controlled gas mixture of C_4_F_8_/SF_6_ at flow rates of 90 sccm/30 sccm respectively and the ICP and RIE power were set to 600 W and 15 W respectively at a chamber pressure of 15 mTorr.

### Characterizations

Surface morphologies were imaged by scanning probe microscopy (SPM, Park systems, XE-100) in tapping mode and scanning electron microscopy (SEM, FEI Company, FEG Quanta 6700 and Zeiss Ultra Plus). The film thicknesses were measured by optical ellipsometer (Woolam M2000) and electron microscopy. Samples were prepared for TEM cross sectional imaging with an FEI Helios Nanolab 600i system containing a high resolution Elstar™ Schottky field-emission SEM and a Sidewinder FIB column and were further imaged by transmission electron microscopy (TEM, JEOL 2100 and an TEM, FEI Titan system). X-Ray photoelectron spectroscopy (XPS) experiments were conducted on a Thermo K-alpha machine with Al K_α_ X-ray source operating at 72 W.

## Electronic supplementary material

Supporting Information
