# Supplementary material for: Development of Ordered, Porous (Sub-25 nm Dimensions) Surface Membrane Structures Using a Block Copolymer Approach
Source: Sci Rep. 2018 May 8;8:7252. doi: 10.1038/s41598-018-25446-0 (PMC5940818; doi:10.1038/s41598-018-25446-0)
Supplement: Supplementary file 1 — Supporting Information [file 41598_2018_25446_MOESM1_ESM.pdf]

Supporting Information:

Development of Ordered, Porous (Sub-25 nm Dimensions) Surface Membrane Structures Using a Block Copolymer Approach

Tandra Ghoshal,[1*] Justin D. Holmes,[1] Michael A. Morris[2*]

The effects of PS/PEO ratio on microphase separation of the PEO-PS film

The effects of microphase separation process and the surface morphologies by varying the PS/PEO ratio is studied by blending the BCP with either PS homopolymer (mol. Wt. 16000) or PEO homopolymer (mol. Wt. 40000). The PEO-PS and the homopolymer were dissolved in toluene in 9:1 ratio to make 1wt% polymer blend solution which was further used for spin coating and solvent annealing. (Figures S1a and b) shows the AFM images of the PEO-PS films with the addition of PS and PEO respectively after solvent annealing in Chloroform at 60 °C for 30 min. In addition of PEO, microphase separated hexagonal PS cylinders were formed inside the PEO matrix in most of the areas; also pattern degradation noticed (Figures S1a). In contrast, both perpendicular and parallel orientated PS cylinders formed in addition of PS (Figures S1b). This might be due to more exposed surface area of PS microdomains.

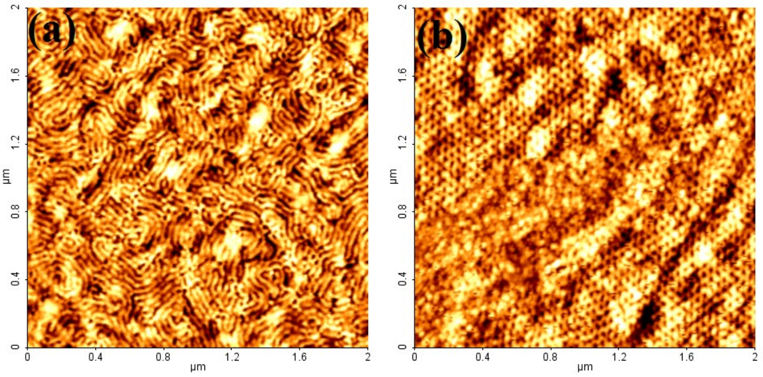

Figures S1: AFM images of the PEO-PS films with the addition of (a) PS and (b) PEO respectively after solvent annealing in Chloroform at 60 °C for 30 min. Images represents 2 x 2 µm area.

Iron oxide formation using ethanol as a precursor solvent

(Figure S2) represents the SEM image of the iron oxide nanopatterns prepared using the BCP nanoporous templates with 0.3 wt% precursor-ethanolic solutions. Ethanol causes undulation, pattern degradation and swelling between nanopores over the wafer scale area.

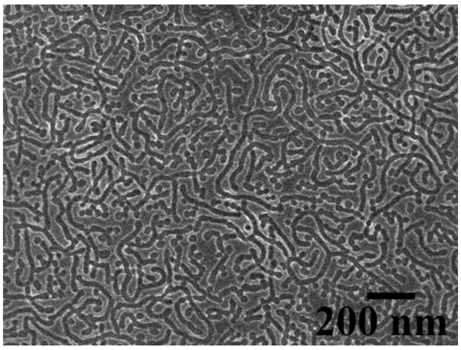

Figure S2. Iron oxide nanopatterns using ethanol as a precursor solvent.

Effect of precursor concentration

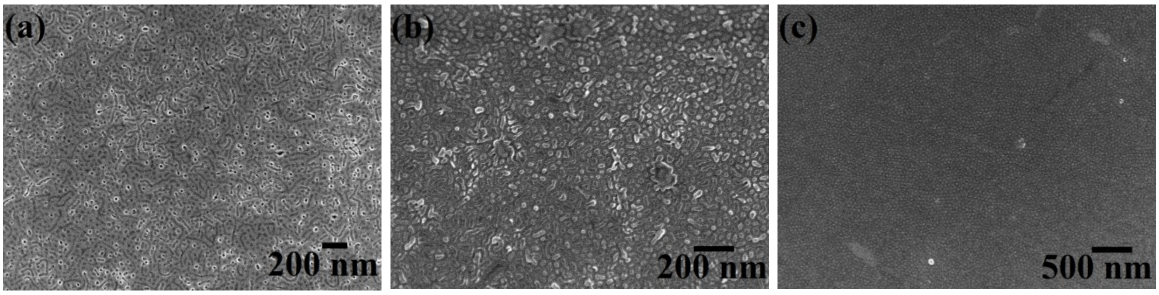

Figure S3. SEM images of the iron oxide nanopatterns prepared using the BCP nanoporous templates with (a, b) 0.6 wt% and (c) 0.1 wt% precursor-2 propanol solutions respectively.

The concentrations of the precursor solutions were tuned to form uniform, continuous and smooth nanoporous patterns. (Figures S3a-c) represents the SEM images of the iron oxide nanopatterns prepared using the BCP nanoporous templates with 0.6 wt% and 0.1 wt% precursor-2 propanol solutions respectively. Higher concentrations lead to overfilling of the pores (Figure S3a) and secondary overlayer formation (Figure S3b) whereas less concentration forms discrete nanopores (Figure S3c).
